# Justice for Women After Sexual Assault: A Critical Interpretive Synthesis

**DOI:** 10.1177/15248380241248411

**Published:** 2024-05-09

**Authors:** Joanna Collaton, Paula Barata, Mavis Morton, Kim Barton, Stephen P. Lewis

**Affiliations:** 1 University of Guelph, ON, Canada

**Keywords:** sexual assault, reporting/disclosure, support seeking, prevention, intervention

## Abstract

Justice after sexual assault is often understood and enacted through the criminal legal system such that the outcomes are binary (i.e., justice is achieved or not achieved). Previous research indicates that survivors have specific wants and needs following an assault in order to experience justice, which may or may not align with current practices. We conducted a critical interpretive synthesis of 5 databases to create a sampling frame of 4,203 records; the final analysis included 81 articles, book chapters, and policy documents. Results indicate that justice is an individualized and dynamic process which may include the experience of voice, connectedness, participating in a process, accountability, and prevention. The experiences of safety and control are central to each of these domains. Survivors may seek and enact these justice domains through several avenues, including the criminal justice and legal systems, restorative justice, medical/mental health spaces, activism, art, and social media. Existing actors within currently available justice systems, including legal, medical, and mental health personnel should encourage survivors to identify and define their own experience of justice, including locating helpful behaviors rooted in safety and control, and resist a binary model of justice. Extant systems should therefore be flexible and accessible to help survivors realize their preferred modes of justice.

Interpersonal violence against women, including sexual assault and rape, is common, with one in four women experiencing physical and/or sexual violence in their lifetime (World Health Organization, 2021). Sexual assault is associated with high rates of posttraumatic stress, anxiety, depression, low self-esteem, sexual disorders, relationship difficulties, substance use, and suicidality (Campbell et al., 2009; Dworkin et al., 2017; Khadr et al., 2018). Although many women experience sexual assault, intersecting systemic and sociopolitical factors of oppression and marginalization (e.g., racism, colonialism, sexism, homophobia, transphobia, ableism) put particular groups of women at higher risk of sexual assault (Conroy & Cotter, 2017; Ogrodnik, 2010). The impact of trauma is multi-faceted, including both the traumatic event and those that follow, which can include disclosures, trauma processing, and service utilization, meaning that survivors require a complex set of services available to them (Campbell et al., 2009).

Following an assault, survivors have many intersecting needs and wants that include but are not limited to physical safety, psychological well-being, and justice (Daly, 2017; Draucker et al., 2009; Wager, 2013). Dominant paradigms in clinical, legal, and research contexts often conceptualize justice and healing using a deficit or symptom-reduction model (e.g., once a survivor’s symptoms abate, she is healed) or as a binary outcome (e.g., she has or has not achieved justice via legal means). Social understandings of justice are limited to this binary outcome based on the procedural steps a survivor takes in criminal legal proceedings (e.g., reporting the event and participating in a trial; Martins Vasconcelos Senra, 2021; McGlynn & Westmarland, 2019). Yet, this paradigm has been challenged by process- and staged-based models of justice and healing (e.g., kaleidoscope justice; McGlynn & Westmarland, 2019, Victims of Violence Program; Herman, 2015), as the former is limited in scope. Indeed, many survivors seek alternative forms of justice outside of the legal system, including restorative justice, activism, art, truth and reconciliation commissions, public naming of abusers, and public apologies (Powell, 2015). Seeking alternative forms of justice is due to general dissatisfaction with the criminal legal system (Lorenz et al., 2019), despite rape law reforms (see Levine, 2018 for an overview).Scholars have pointed to several overlapping components across models of justice. In Herman’s model (2023), she outlines three phases of justice (acknowledgment, apology, accountability) and healing (restitution, rehabilitation, prevention) but intertwines these when speaking about “healing justice” as an overarching concept. McGlynn and Westmarland (2019) delineate kaleidoscopic justice as comprising consequences, recognition, dignity, voice, prevention, and connectedness. Koss (2010) differentiates between survival needs and justice needs, such that survival may include “safety, physical health, economic [security], including housing and employment, education, or retraining; and [addressing] immigration problems” whereas justice needs “involve an innate motivation to right wrongs” (p. 221). Daly (2017) further differentiates between the concept of justice interests over justice needs. Accordingly, Daly purports that justice interests include participation, voice, validation, vindication, and offender accountability-taking responsibility. She also differentiates safety, coping/healing, and justice, such that they are distinct and distinguishable constructs. However, Wager (2013) combines justice and healing toward re-establishing safety via accountability, having questions answered, relational repairs, including validation and vindication, and self-restoration. Taken together, there are overlapping constructs regarding justice conceptualizations. Definitional clarity across relevant constructs as well as where, when, and how survivors enact them can clarify how to provide justice-oriented opportunities commensurate with survivors’ wants and needs.

Much research has indicated that survivors are unsatisfied with current procedural offerings for them, particularly in the criminal legal system, which can result in secondary victimization (Campbell, 2014; Campbell et al., 2009). To provide appropriate services that meet survivors’ needs and priorities, it is thus important to wholly understand their justice needs and wants, both within and beyond the current systems in place. Specifically, it would be helpful to move beyond a procedural level (i.e., satisfaction with the criminal justice system) and instead investigate a more nuanced definition of justice that focuses on understanding its’ underpinnings (i.e., what comprises justice and where survivors find it). Because the definition of justice appears idiosyncratic, a critical interpretive synthesis (CIS) of the literature seems warranted.

CIS is a knowledge synthesis method appropriate for research questions with broad and heterogenous literature (e.g., qualitative, quantitative, mixed-methods research as well as other reviews, theory papers, and commentaries found in peer-reviewed and gray literature; Dixon-Woods et al., 2006). It is a flexible approach to knowledge synthesis as an alternative to a systematic review, which summarizes quantitative data, and meta-ethnography, which synthesizes qualitative information. Unlike other approaches, CIS is an iterative, dynamic, and reflexive process of knowledge synthesis that involves searching and sampling the literature, assessment of inclusion via appropriateness rather than quality, as well as components of traditional meta-ethnography (Noblit & Hare, 1988). Its aim is to generate theory with an emphasis on critical engagement with extant literatures. In this way, a CIS differs from a narrative review which is often a qualitative summary of a topic without the reflexive and interpretive involvement of the researcher.

A CIS focused on how women understand justice after sexual assault represents one step toward better-understanding women’s post-assault experiences by developing a more fulsome understanding of how women conceptualize justice. It is particularly important to understand justice beyond interactions with the criminal justice system, as much of this research is embedded within this domain. Understanding the comprehensive justice wants and needs of survivors is a challenge; a literature search for “sexual assault,” “justice,” and “wants and/or needs” returns hundreds of thousands of articles and books on the topic. Given this vast and complex literature, a CIS may be a potent, rigorous, and feasible approach to summarize information, as it permits synthesis of the literature using a critical framework which allows the researcher to question and critique the literature base as a whole. Such an approach may help (re)conceptualize justice, provide new insights, and establish questions for future research.

There have been a few literature reviews conducted on women’s experience of justice, including on justice for older women (Fileborn, 2017b) and the impact of race on criminal legal responses to sexual assault (Shaw & Lee, 2019). There have also been two reviews on restorative justice (Cheon & Regehr, 2006; Donaghy, 2020), which is broadly understood as a transformative approach which may involve mediation meetings with a survivor and their perpetrator; the focus of the meetings is on the “repair of harms and of ruptured social bonds” (Daly & Immarigeon, 1998, p. 21). Although restorative justice has, in some ways, dominated understandings of justice in recent years, it is only one way of looking at justice, and as such, we were interested in a more fulsome review of what justice is and how it can be achieved.

As well, these reviews are narrower in scope and use different methodologies than we conducted herein. A systematic review was not appropriate because we wanted to understand justice using an evolving definition according to the literature we reviewed and because our goal was to integrate multiple literatures, critique the literature base as a whole, and generate a theory of justice. There are currently no reviews adopting a CIS approach to critically synthesize the literature to generate a theory of justice for survivors of sexual assault. Therefore, a CIS was conducted to investigate what justice means to women after sexual assault.

## Method

CIS comprises six phases: generating a flexible research question, searching the literature, sampling and selecting literature, data extraction, quality appraisal, and formulation of synthesizing arguments and synthetic constructs (Depraetere et al., 2021; Dixon-Woods et al., 2006).

### Searching and Sampling the Literature

#### Purposive Sampling

Purposive sampling can be done in many ways, with the goal of ensuring the richness of information at the initial stages (Ames et al., 2019). We employed a snowball technique, including initial trial searches related to justice and sexual assault, followed by a review of references of relevant articles. This generated a preliminary list of 17 studies to inform the subsequent phase of sampling (Dixon-Woods et al., 2006). This list included gray literature, book chapters, and journal articles. Articles were selected during this phase if they addressed models of justice or described the wants and needs of women survivors of sexual violence. The following data were extracted: Authors, Title, Year, Context and participants, Study design and Methods used, and Findings (Noye et al., 2022).

Next, the following concepts, which are understood as preliminary synthetic constructs in CIS, were generated by compiling common and unique components of justice from the preliminary list of studies: *voice, participation, accountability, validation, being acknowledged, finding safety, feeling empowered, being in control, prevention, feeling connected to others or finding community, being informed and educated of options, being vindicated, consequences for the perpetrator, being respected and treated with dignity.*

#### Theoretical Sampling

A sampling frame was subsequently generated by searching the literature using the above preliminary synthetic constructs (e.g., *voice, participation*, *accountability*, et cetera) combined with terms related to sexual assault (i.e., sexual violence, sexual assault, sexual abuse, rape) and women (i.e., women, girl, female). This was an iterative process of refining and searching the following databases: PsycINFO, GenderWatch, Sociological Abstracts, CINAHL, and ERIC. Search terms were trialed, and some were removed as they generated considerable noise (e.g., “control” and “validate”). The search was limited to papers in English. In consultation with a librarian, we searched using the title and keywords only, thus prioritizing a specific versus sensitive search. This decision was made based on feasibility and our aim to identify highly relevant articles to establish a sampling frame rather than conduct a comprehensive search. A research assistant also identified and hand-searched publications from government and violence against women-related websites for gray literature (Appendix A) using the preliminary synthetic construct terms.

The final search included the following terms and parameters: TI,AB (“sexual violence” OR “sexual* abuse*” OR “sexual* assault*” OR rape OR rapes OR raped) AND TI,IF (justice OR voice OR “story telling” OR silence OR hear* OR recognize OR recogniti* OR validat* OR acknowledg* OR ally* or allies OR help* OR assist* OR retribution OR vindicat* OR punish* OR “community safety” OR prevent* OR rehab* OR accountability OR secur* OR activis* OR heal OR healing OR empower* OR power OR control* OR choice* OR participat* OR inform*) AND TI,AB (women OR woman OR girl* OR female*). This search was conducted in January 2022 with no specific time limitations.

**Figure 1.**
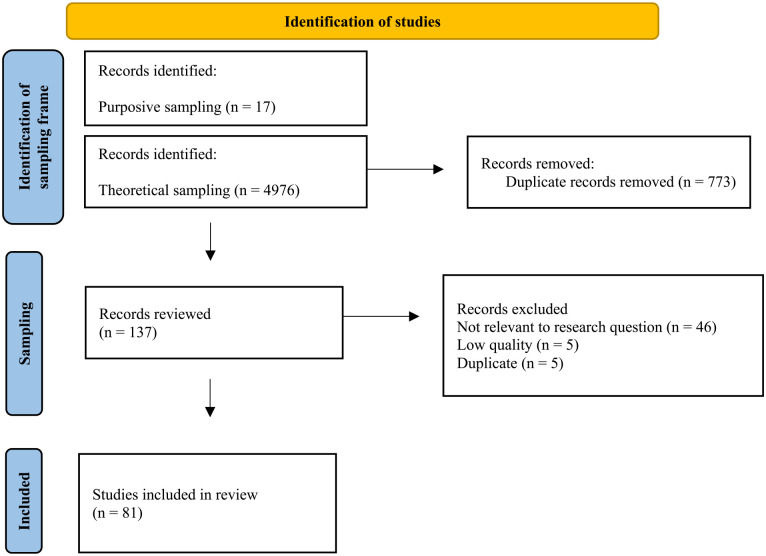
Identification of studies.

The resultant records were combined (*n* = 4,976). Deduplicating using Mendeley resulted in a sampling frame of 4,203 records from which to review and extract data. Based on the iterative and dynamic approach to CIS, we did not have a priori inclusion or exclusion criteria; rather, this shifted throughout the sampling phase as is standard practice in CIS (Depraetere et al., 2021; Dixon-Woods et al., 2006). The sampling and extraction process was informed by qualitative methodologies such as theoretical sampling (Glaser & Strauss, 1967) and conceptual depth, an alternative to saturation. For example, as our understanding of voice as justice changed, so did the way that we read each subsequent article; at the sampling onset, our understanding of voice was limited to a disclosure process, although this shifted throughout the analysis to include silence and engaging in arts-based practices. Consequently, this sometimes required looking for particular concepts within the sampling frame based on our emerging understanding of voice to clarify the bounds of each synthetic construct and distinctions between constructs (e.g., between voice and connectedness). This process yielded 137 records. Figure 1 depicts how studies were identified.

### Data Extraction

From the 137 records, data were extracted from 81 records using the same standardized form for the initial sampling phases (note that these records are denoted by an asterisk in the reference section). Of these 81 records, they were broken down into the following methodologies: qualitative (*n* = 33), quantitative (*n* = 18), mixed methods (*n* = 5), books (*n* = 1), commentaries or newspaper articles (*n* = 8), reports or working papers (*n* = 4), theoretical papers (*n* = 5), and program development or evaluation articles (*n* = 2).

### Quality Appraisal

Unlike systematic reviews, quality appraisal is not a focus of CIS as the goal is to integrate and synthesize studies of various methodologies, theories, commentaries, and reviews, which do not have a hierarchy of quality, unlike quantitative studies (Dixon-Woods et al., 2006). Instead, JC and KB discussed each extracted article at weekly meetings to assess their appropriateness and quality. If a study did not answer any part of the research question (i.e., what does justice mean to women after sexual assault? (*n* = 33) or was fatally flawed according to criteria used by Dixon-Woods et al. (2006) (*n* = 5; e.g., the researchers presented insufficient data to support interpretations and conclusions), the study was excluded. Five duplicates were also removed. Disagreement between authors was resolved by speaking to a third study team member. Weekly meetings between JC and KB ensured that we developed a critical review that was not of a singular perspective and allowed us to discuss areas of convergence, divergence, and critiques of the evidence base as a whole.

### Formulation of Synthesizing Arguments and Synthetic Constructs

Data extraction was terminated when we reached conceptual depth (Nelson, 2017). Conceptual depth rather than saturation was chosen because of notable issues with the concept of saturation, including that there are always new things to discover in data and that in some research questions, there are potentially limitless themes and examples (see O’Reilly & Parker, 2013). We believed this to be true of our research question, based on the individualized approach to justice that emerged from the literature. As such, to reach conceptual depth, we were looking for a large scope of evidence to demonstrate the concepts, an interconnected network of complex connections between concepts, an understanding of the concepts and their subtle differences, and integration into the extant literature with external validity (Nelson, 2017). Once data extraction was completed, JC met with KB to organize the information and ensure agreement on the final analysis.

Regarding our positionality, JC came to this analysis with a frame of lived experience as both a survivor and a witness to the justice and healing journeys of peers and clients, and as a training clinical psychologist. She has also been learning in feminist spaces, both academic and community, for the last 12 years. Hence, her reading of the literature is informed by her experiences as well as the privileges she holds. She is a cis, white, settler woman of North American citizenship who has benefited greatly from the feminist community, therapeutic processes, and academic opportunities that have been available to her. She uses a social constructivist and feminist epistemological approach, believing that women’s experiences are socially bound by the world in which we live. The project’s research assistant, KB, came to this research grappling with the identity of survivor. As a cis, white, settler woman working within academic and community leadership, she identifies as a privileged Registered Early Childhood Educator and is indebted to the women and femme educators, carers, healers, academics, and advocates before her. At the time of this project, her interpretations of the literature were informed by an ethos toward generating knowledge about mutual flourishing and healing, learning, and living well throughout the human experience.

## Results

### What is Justice After Sexual Assault and How Is It Enacted?

**Figure 2.**
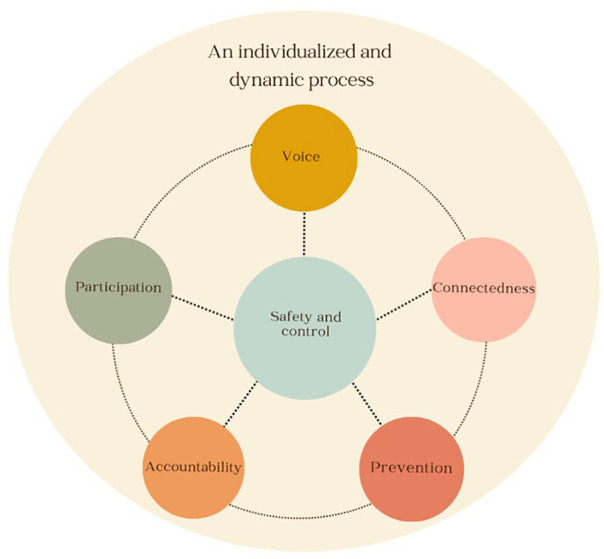
Justice model illustration.

Justice can be understood as being composed of five underlying synthetic constructs, including having a voice, accountability for the perpetrator, participating in a process, being connected to the self and others, and prevention of future harm. These synthetic constructs interact and intersect for many survivors over time with shifting wants and needs throughout their lifespan. They are not mutually exclusive entities, and many survivors experience multiple aspects of justice simultaneously through one activity (e.g., a survivor can enact voice and seek connectedness when disclosing to others). Each synthetic construct is rooted in the concept of safety and control, meaning that survivors value agency and safety *within* each synthetic construct (described further below). For this manuscript, the constructs have been written about in serial order, although it is important for the reader to conceptualize them as nonlinear, process-based, and intersecting. See Figure 2 for an illustration of this model.

#### Justice as an Individualized, Dynamic Process

Justice can be understood as a nonlinear, dynamic, and individualized process (Decker et al., 2020; Keenan, 2014; McGlynn & Westmarland, 2019). McGlynn and Westmarland (2019), informed by the work of Daly (2014) and others (Clark, 2015; Herman, 2005; Holder, 2015; Jülich, 2006), propose a theory of kaleidoscopic justice, understood as “a lived, ongoing and ever-evolving experience and process, rather than an ending or result” (p. 186). In this way, as the survivor must continually exist with the impact of the sexual assault, so too is her journey for justice a continual, lifelong process. The authors use the metaphor of a kaleidoscope to demonstrate that justice needs and wants are ever-changing and often shifting (McGlynn & Westmarland, 2019). In line with this, Clark (2015) argues that justice, as an endpoint, is not achievable as the act of sexual violence cannot be undone. Interviews and focus groups with survivors indicate heterogeneity among respondents when asked about what justice means to them; one survivor explained aptly that “it depends on the person and the situation” (Decker et al., 2020, p. 6). This notion was paralleled by participants in another study, such that justice “depends on the individual” (McGlynn & Westmarland, 2019, p. 194). Richards et al. (2021) further emphasize that survivors’ views of justice, and the associated criminal legal response, are variable, multi-dimensional, and dynamic. As such, justice can be understood as varying between and within individuals over time.

#### Justice as Having A Voice

##### What Voice Can Mean

Many authors reported the importance of survivors having the freedom to speak about their experiences and an opportunity to share their stories (Keenan, 2014; McGlynn & Westmarland, 2019), including their content and impact (Daly, 2014). In some cases, this might involve survivors sharing their stories in a significant setting (e.g., to family or a jury) that is deemed safe to them (Daly, 2014; Jülich, 2006). McGlynn and Westmarland (2019) describe voice as a process of speaking out and making sense of the harm they experienced in a way that is heard by perpetrators, family members and/or friends and allies. As such, voice often implicates active listening from others and is thus closely connected to the experience of *connectedness* (see below).

##### Where Survivors Find Voice

There are many different settings survivors find a space for voice. With respect to formalized processes, many survivors cite the role of speaking out as part of their desire to report, as well as being informed and having a say in the legal process (Hawkins & Laxton, 2014; Jülich & Landon, 2017; Kelly, 2011; McGlynn & Westmarland, 2019; Mulvihill et al., 2018; Wager, 2013). Specifically, survivors may tell their assault stories to police, medical or mental health professionals, or clergy members (Lorenz et al., 2019; Starzynski et al., 2005; Ullman & Peter-Hagene, 2014; Young et al., 2018). Survivors also find voice in less formal settings in their personal lives with friends and family (Starzynski et al., 2005; Ullman & Peter-Hagene, 2014).

Additionally, survivors may find voice in a setting that encourages the use of art and non-verbal communication as part of storytelling. For example, Kigvamasud’Vashti et al. (2011) describe a documentary film and subsequent screening of “NO!,” a film centered on sexual violence against Black women, as an opportunity for voice. Specifically, developing the film provided opportunities for survivors to share their stories and for the director, Aishah Shahidah Simmons, who identifies herself as a survivor of rape, to explore her own experience and find voice as part of this project. Screenings of the documentary also acted as potential avenues for voice, as they were organized to facilitate “transformative dialogue” (p. 61) for viewers. Survivors also have engaged with art installations to enact voice. For example, in 2015, a Kosovan artist named Alketa Xhafa Mripa created an art installation titled “Mendoj Për Ty,” meaning “Thinking of You” which consisted of 5,000 dresses (Di Lellio et al., 2019). The installation was dedicated to survivors of sexual violence from the Kosovo War, as it gave voice to a wartime secret. In a similar vein, Herman (2023) talks about a community activist, Sarah Super, who advocated within her local community to erect a monument for survivors of sexual assault in her town as a way to give voice to her and other survivors’ experiences.

A whisper network is a long-used form of sharing information that came to the forefront most recently as part of the #MeToo movement. Examples of this include the Shitty Media Men list (Corcione, 2018) and #LoSha, a list of sexual harassers in Indian academia (Gajjala, 2018). The advent of the Internet has allowed the widespread sharing of these lists; however, women have been sharing names of abusers for much longer than this. For instance, in 1990, a young woman who was date raped wrote the name of her abuser on the bathroom wall of her university library; the list quickly grew to over 30 names and prompted widespread discussion of sexual assault policies on campus (Celis, 1990).

Survivors also take part in activism and online engagement as a means of voice. Qualitative interviews with survivors involved in antiviolence activism found that engaging in activism promoted feelings of empowerment and freedom, in part due to gaining comfort publicly speaking about their experiences with sexual assault (Strauss Swanson & Szymanski, 2020). This is also reflected in online spaces, where survivors can have digital voices on Tumblr (Loney-Howes, 2015), Reddit (O’Neill, 2018), or mental health peer support apps (Collaton et al., 2022). In qualitative surveys and focus groups, survivors have shared that online discussions about their experiences with street harassment gave them a sense of permanence, catharsis, and a space to vent (Fileborn, 2017a). Powell (2015) discusses online spaces as an avenue for disclosure and story sharing and as an opportunity to seek redress. She frames online disclosures as an opportunity for a testimonial, as well as to receive advice and support from community.

Beyond activism, survivors may also find voice within a therapeutic relationship, including individual, group, and arts-based therapy. This has been demonstrated across age groups, including children (Capella & Boddy, 2021), adolescents (Backos & Pagon, 1999; deYoung & Corbin, 1994), and adults (Amir, 2004; Anderson & Gold, 1998; Sweig, 2000; Wright & Thiara, 2019), as well as with varying modalities, such as psychoeducation, group therapy, music therapy, and creative writing. Similar parallels have been made in the process of research participation for survivors (Kirkner et al., 2019).

#### Justice as Connectedness

##### What Connectedness Can Mean

Connectedness as part of justice exists at individual and social levels. Individually, connectedness can mean reintegrating trauma into oneself and feeling whole again (McGlynn & Westmarland, 2019). In this way, connectedness appears to overlap with how survivors understand healing (Anderson & Gold, 1998; Dahlgren et al., 2020). Closely connected to voice is the experience of connectedness with others. This can mean connecting with individuals who have a shared lived experience or supportive allies (Ullman & Peter-Hagene, 2014). Justice as connection can also include receiving validation from others, being listened to, having others bear witness to one’s experience, and acknowledging one’s experience and the harm caused (Herman, 2005; McCulloch et al., 2021; McGlynn & Westmarland, 2019; Strauss Swanson & Szymanski, 2020). For example, survivors in one study indicated a wish for acknowledgment from the perpetrator himself but also validation from family and community members (Herman, 2005).

##### Where Survivors Find Connectedness

Within the self, integration of the trauma is often described as an outcome or goal of a therapeutic process (Backos & Pagon, 1999; Dahlgren et al., 2020). For example, in a group therapy setting for adolescent sexual abuse survivors, group facilitators guided participants toward integration via arts-based practices (Backos & Pagon, 1999). Similarly, Reichert (1994) described targeting integration to loosen defense mechanisms in group therapy for school-aged girls who had experienced sexual abuse.

Connectedness also occurs between survivors and others. This can be experienced as validation from others and can be sought out in many avenues and systems, often mirroring spaces where survivors choose to enact their voices. Survivors may seek the experience of connection when they disclose to formal or informal supports (Starzynski et al., 2005), including friends, family, helplines, or authorities (McGlynn & Westmarland, 2019; Young et al., 2018). Within oft-used systems, survivors may seek and receive validation and connectedness from offenders (Jülich & Landon, 2017; Keenan, 2014; McGlynn & Westmarland, 2019; Wager, 2013), healthcare providers (Munro-Kramer et al., 2017; Rajan et al., 2020), sexual assault response personnel (Henninger et al., 2020; Scoglio et al., 2021), or through an individual or group therapeutic process (Amir, 2004; Anderson & Gold, 1998; deYoung & Corbin, 1994; Fruzzetti, 2017; Sweig, 2000; Wright & Thiara, 2019). In restorative justice models, there are efforts to keep the community intact due to the recognition that harm to one is equivalent to harm to the whole community (Kelly, 2011). As such, these processes are positioned to maintain connectedness between the survivor and their community, which may include the perpetrator. However, this needs to be balanced with the social pressures to reconcile with perpetrators (e.g., in Native American communities; Smith, 2011).

Connectedness can also exist in activist and art spaces (Di Lellio et al., 2019; Strauss Swanson & Szymanski, 2020) and is postulated to foster empowerment (Anderson & Gold, 1998; Strauss Swanson & Szymanski, 2020), which is important for acquiring agency. Virtual connection is another avenue survivors may seek. As above, many survivors disclose their experiences with sexual assault online (Fileborn, 2017a; Loney-Howes, 2015; O’Neill, 2018; Powell, 2015) and, within that context, receive validating and supportive messages in return. The #MeToo movement is a recent example where survivors connected online to share messages of support (Drewett et al., 2021). Through experiences of connection and validation from others, some survivors may learn to validate their own experiences and counter internalized blame. Finally, survivors may seek connectedness with nature and the non-human world, which reduces feelings of isolation (Moore & Van Vliet, 2022).

#### Justice as Finding Accountability

##### What Accountability Can Mean

Daly (2014) defines accountability as “perpetrators taking active responsibility for the wrong caused, to give sincere expressions of regret and remorse, and to receive censure or sanction that may vindicate the law and a victim” (p. 388). Keenan (2014) differentiates between two forms of accountability, namely (a) admission, which requires the perpetrator to admit wrongdoing and to take responsibility for harm caused, and (b) acknowledgment/acceptance, wherein “the offender comes to understand the harm done to the victim and the ripple effect of his crime on those who have been harmed, including himself” (p. 153). In interviews with sexual assault response personnel, respondents indicated that accountability varies between survivors and depends somewhat on the relationship between the survivor and perpetrator (Scoglio et al., 2021). Adult survivors of child sexual abuse similarly noted the role of accountability, which may include a sincere apology and admission in front of others as well as acknowledgment that the act was wrong (Jülich, 2006). While some survivors wish for or seek acknowledgment from the perpetrator, this is not always the case (Herman, 2005; Jülich, 2006; McCulloch et al., 2021).

##### Where Survivors Find Accountability

Survivors access accountability in many justice spaces, including through criminal and restorative justice models that lead to consequences, institutional mechanisms, community-based abolitionist initiatives, and through online discourse and social media. Interviews with survivors have indicated that they seek accountability via public confessions, alimony, child support, donations to rape crisis centers, incarceration, apologies, and intervention programs (Cramer, 2004; Herman, 2005; McGlynn & Westmarland, 2019). In focus groups with intimate partner and sexual violence survivors, participants highlighted accountability through consequences as part of justice, whether that be facilitated via the criminal legal system or on a personal level (Decker et al., 2020). In a different focus group of sexual violence survivors, participants identified personal responsibility as important to accountability, and in preventing future harm (McGlynn & Westmarland, 2019). There are mixed findings regarding revenge and personal punishment, including ambivalence among survivors about whether they want their perpetrator to suffer (Clark, 2015; Herman, 2005). Restorative justice and circles of support and accountability, a quasi-restorative approach focused on reintegrating perpetrators back into the community, also serve as an accountability measure (Jülich & Landon, 2017; Kelly, 2011; Richards et al., 2021).

When a perpetrator and survivor have a shared community, there are avenues for accountability within these spaces. For example, if they attend school together, perpetrators may face sanctions or suspensions (Camp et al., 2018; Reingold & Gostin, 2015). In a shared workplace, employees may be reprimanded via grievance processes and sanctions (Mcdonald, 2012). Online, apps have also been created to report sexual harassment, therefore offering another avenue for accountability (as well as voice and prevention). For example, HarassMap was created in 2010 for individuals in Egypt as a space for anonymously reporting experiences of sexual harassment (Peuchaud, 2014). Survivors also use the internet and social media to seek redress when sharing their stories (Powell, 2015). Powell frames this action as “feminist response-ability,” which can be understood as integrating voice, connectedness with community, and accountability. The #MeToo movement and subsequent “naming and shaming” of abusers emerged from a lack of accountability across justice spaces but can also be understood as an accountability strategy employed by survivors (Berndt Rasmussen & Olsson Yaouzis, 2020).

Discussions of accountability also include community accountability. Kim (2011) describes working toward accountability from a community and abolitionist framework that actively resists individualized, neoliberal approaches that prioritize criminalization and the difficulties that follow this work. For example, after a sexual assault occurred within a Korean drumming community, the group came together to establish workshops, improve training for all members, add more women to leadership positions, and advocate for therapy and a leave of absence for the perpetrator. Based on this experience and the difficulty this group had locating other examples of similar processes, another project, StoryTelling and Organizing Project (STOP), emerged. STOP shared stories of community accountability alternatives, with reflections on the successes and pitfalls, as an approach to inspire like-minded, progressive groups seeking justice processes that better fit their values (Kim, 2011). This group reflected on the differences between accountability and consequences and the difficulty in moving past the role of punishment based on collective and social ideas of what accountability means (Kim, 2011). Kigvamasud’Vashti et al. (2011) similarly discussed community accountability during post-screening discussions of the film “NO!,” described earlier. With respect to accountability, the authors point to the use of documentary filmmaking and post-screening discussions as spaces for community building and brainstorming for alternatives to reliance on police in accessing accountability after a sexual assault (Kigvamasud’Vashti et al., 2011).

#### Justice as Participating in A Process

##### What Participating Can Mean

Participating in a formalized process is often understood as engaging in a procedural justice setting, including being informed of the options and developments in a case and types of justice mechanisms available, as well as taking an active role in the direction of the process (Daly, 2014; Decker et al., 2020; Jülich, 2006; Keenan, 2014; McGlynn & Westmarland, 2019). Some researchers understand participation and voice as overlapping or indistinguishable phenomena in that to have a voice is to have some control over the justice proceedings (McGlynn & Westmarland, 2019). Being informed of the process promotes empowerment and safety (Hawkins & Laxton, 2014). As such, Cramer (2004) argues that survivors should be given realistic information about legal options, thus augmenting the sense of having choice.

##### Where Survivors Can Participate

Survivors *participate* in many settings. In criminal legal proceedings, survivors report the crime and in doing so may provide statements, answer questions, provide physical evidence, and participate in an investigation where they make multiple disclosures to officers and detectives (Patterson & Campbell, 2010). In a review, Mulvihill and colleagues (Mulvihill et al., 2018) describe almost 20 theoretical models of justice and the ways that they overlap and differ. They highlight a few models that promote greater participation, as defined above, to include high levels of being informed and having agency and control, such as participatory jurisprudence (in which participants are supported in communicating and participating actively) and therapeutic justice (in which there is a focus on how participating in a legal process can impact the survivors’ mental health; Mulvihill et al., 2018).

Restorative justice has also been a major focus of study. This model aims to repair harm toward rehabilitative ends; it may include a conferencing process whereby a survivor, perpetrator, and/or organization participate (Jülich & Landon, 2017). This process has been integrated into the criminal legal system as a diversion route but has also been implemented in community groups. The RESTORE program has been used in the United States (Koss et al., 2003) and New Zealand (Jülich & Landon, 2017) as diversion tactics put in place as an option for survivors. In RESTORE’s first iteration, survivors and perpetrators went through three stages: preparation, conferencing, and accountability/supervision. The program has demonstrated efficacy in that restorative justice is a feasible, safe, and satisfying approach for many survivors (Koss, 2014). In addition to formalized programs, grassroots work has been implemented that does not implicate the legal system. Kelly (2011) describes the work of two community groups, Philly’s PISSED and Philly Stands Up, that came together to implement a restorative justice model within their community of anarcho-punk folks after a series of sexual assaults in 2004. Over time, this work resulted in downstream effects on the group, such as creating a culture of sexual responsibility. Like the above-described restorative approaches of the Korean drumming community (Kim, 2011), this work highlights the close linkage between the role of accountability and how survivors access accountability via participation.

Additionally, other settings have been postulated to provide avenues for participating in a process following the experience of sexual assault. Namely, Loney-Howes (2015) describes engaging in anti-rape activism and organizing, particularly participating in social media campaigns such as Stop Rape Now, This is Not an Invitation to Rape Me, and Project Unbreakable, as ways for survivors to participate in a justice process. Powell (2015) similarly points to the advent of social media as a positive way to increase the spaces in which survivors may participate in informal justice processes.

#### Justice as Prevention

##### What Prevention Can Mean

Prevention, in short, is not wanting the act of sexual assault to occur again either to themselves or to other people (Clark, 2015; Herman, 2005; Keenan, 2014; McGlynn & Westmarland, 2019; Patterson & Campbell, 2010; Scoglio et al., 2021; Wager, 2013). In this way, prevention is sometimes closely linked with accountability, as this is a motivating factor for reporting (Patterson & Campbell, 2010; Richards et al., 2021). As some survivors express concerns about the efficacy of reporting the event as a crime and potential subsequent incarceration, many survivors also wish for higher-level change, such as shifting sociocultural attitudes about rape culture and violence against women (Clark, 2015; Decker et al., 2020; McGlynn & Westmarland, 2019). Education appears to play a big role in prevention efforts for survivors (McGlynn & Westmarland, 2019), as well as addressing the underlying motivations that perpetuate sexual violence (Jülich, 2006). Prevention can thus exist across primary, secondary, and tertiary levels, with education being central to each.

##### Where Survivors Can Work Toward Prevention

Individually, survivors often engage in tertiary prevention practices to prevent further harm. For example, survivors engage with police and other criminal justice actors as a safety mechanism to protect themselves and prevent additional harm (Clark, 2015; Patterson & Campbell, 2010; Wager, 2013). Restorative models may also serve to prevent future harm (Kelly, 2011; Smith, 2011). Survivors may also seek out healing or therapeutic processes (Decker et al., 2020), which can be seen as to prevent future harm by working to change intrapsychic and relational factors that may contribute to continued involvement in abusive relationships (Ørke et al., 2021; Reichert, 1994).

As survivors outline the need to change sociocultural attitudes about rape culture and deconstruct patriarchal systems (Clark, 2015; Decker et al., 2020; McGlynn & Westmarland, 2019), there is a large body of work dedicated to delineating avenues to do so with the potential for involvement from people with lived experience. This includes large-scale primary and secondary prevention efforts ongoing across community groups, much of which is mostly concentrated in North American university campuses (Backman et al., 2020; Baldwin-White & Moses, 2021; Cadaret et al., 2021; DeLong et al., 2018; Hahn et al., 2017; Hubach et al., 2019; Jozkowski, 2015; Orchowski et al., 2020; Senn et al., 2013), though some work has occurred in Europe (Camp et al., 2018; Lickiewicz et al., 2021). Evidence from these efforts indicates the need for a comprehensive and mutually reinforcing approach that integrates programming for individuals most likely to be perpetrated against (e.g., self-defense) and perpetrate violence (e.g., education), as well as bystanders via consciousness-raising and bystander intervention (Cadaret et al., 2021; Orchowski et al., 2020). To meet the goals of survivors, this type of programming should consider ways to promote the deconstruction of social factors such as gender roles, media messages, and sexuality including via reflexive practices and group discussion (Jozkowski, 2015). According to survivors, their attendance at university-based programming should be optional, as it can sometimes be triggering or activating (Worthen & Wallace, 2021); this highlights the importance of choice and safety when survivors engage in prevention-based work. Primary prevention strategies include having comprehensive policies regarding campus sexual violence as one component of sexual violence prevention strategies to reduce and prevent campus sexual assault (DeLong et al., 2018).

Other work has been done to fill gaps regarding the experience of women and gender-expansive individuals who may not be represented in on-campus prevention efforts, as they often center experiences of young white women. As prevention efforts tend to work better when culturally tailored, when they address the unique social context in which it is being delivered, and when they involve important actors such as community leaders, peers, and families (Backman et al., 2020; Baldwin-White & Moses, 2021; Hubach et al., 2019; Khoori et al., 2020; Louie, 2018; Tillman et al., 2010; Yu et al., 2017), therein exists further chances for survivors to engage in prevention-related efforts. Further, there have been recent calls for prevention efforts to be more inclusive of gender-expansive and disabled folks (Janse van Rensburg & Smith, 2021) which would open up new avenues for marginalized survivors to participate. As such, there are many opportunities for survivors to enact prevention efforts either through direct involvement, consultation, or policy work within educational and community-based settings.

Activism which includes consciousness-raising, including online engagement, is another means for survivors to work toward prevention. For example, filmmaking has been purported to spread awareness and education about the impact of sexual violence (Banwari, 2015; Kigvamasud’Vashti et al., 2011). Survivors may also engage within activist spaces as a method for education and prevention, as they have expressed finding ways to help others and participate in feminist advances to reduce rape culture and sexual violence (Backos & Pagon, 1999; Strauss Swanson & Szymanski, 2020). This can also occur online via social media and blogging (Fileborn, 2017a; Loney-Howes, 2015).

### Safety and Control

An overarching theme that connected each domain is that of safety and control. Namely, survivors need to experience empowerment and choice of whether they would like to enact voice, connectedness, accountability, participation, and prevention. For example, when speaking out and using her voice, a survivor must feel safe to do so (Daly, 2014; Jülich, 2006). In this way, survivors must have agency of when to speak or stay silent. Chubin, for example, writes about her lived experience as an Iranian woman in Tehran and how her silence can be understood as resistance to street sexual harassment (Chubin, 2014). This conceptualization of voice highlights the importance of choice and safety in justice-oriented activities. When speaking out online, the anonymity of the internet may act as both a safety mechanism and one of danger, such that online spaces can be rife with harassment; thus, online engagement is a complex decision similar to other avenues for voice and participation (Fileborn, 2017a; Powell, 2015).

In seeking connectedness, survivors often disclose their experiences to individuals whom they view as safe, such as friends and family (Starzynski et al., 2005). With respect to accountability, some survivors report it is important to have a say in how their perpetrator is held accountable, thus indicating the importance of control in this domain (Herman, 2005). As survivors participate in justice processes, including criminal and restorative justice, being informed, in control, treated with dignity, and feeling safe are reported as central to continued participation (Donaghy, 2020; Hawkins & Laxton, 2014; McGlynn & Westmarland, 2019). Similarly, survivors report that group therapy and art-based group practices require safety guidelines (Anderson & Gold, 1998; deYoung & Corbin, 1994). Finally, to engage in prevention work, survivors must feel safe and in control. Without this, they may experience burnout, triggers, and overwhelm (Strauss Swanson & Szymanski, 2020). As such, safety and control are closely tied to each justice domain because they appear to be essential to justice (Figure 2).

### Critiques of the Literature as A Whole

We observed a tendency in the literature to maintain the current carceral system and work on improving survivor experiences. To some extent, this focus seemed to work against lived experience perspectives of justice. The proliferation of the criminal legal system and carceral punishment for socially immoral behavior makes it difficult for some survivors to think beyond a carceral response and to view their post-assault actions, which may include keeping their experience to themselves for example, as justice-seeking. Within our review, survivors often expressed conflicting thoughts and feelings about justice, such that they want their abuser to be imprisoned to not harm again, yet do not think incarceration will change him for the better. Yet, there is still considerable work being done to encourage survivors to report their experiences to the police. This results in a clear hierarchy with respect to justice-seeking avenues, with the criminal legal response prioritized as superior. Maintaining this hierarchy may be invalidating to many survivors who, for good reason, choose not to engage with a system that they believe works against them. Moreover, as justice may better be understood as ever-changing, it is difficult to expect one system to meet all survivor needs.

Based on the large emphasis on prevention as part of justice, there have been considerable efforts to reduce rates of sexual violence. Our observation was that much of this work in recent years has focused on addressing rape culture and bystander intervention with young adults at universities in North America; this is due to grassroots advocacy and relevant legislation (Newlands & O’Donohue, 2016; Pietsch, 2022). While important, a by-product of this focus is that much prevention work has historically centered the voice of young, white cis women in North America. We thus specifically sought out research that was more culturally responsive and diverse. Nevertheless, it was difficult to ignore the abundance of work centered on a potentially privileged group of women.

## Discussion

Justice can be understood as a dynamic, ever-changing, individualized process with anchors in safety and control. As part of justice, survivors may seek out and enact voice, connectedness, accountability, participation in a process, and prevention of future harm. Survivors seek out these constructs at varied times and contexts across their lifespan. This includes formal systems, such as the criminal legal, medical, and mental healthcare systems, as well as alternative counter-spaces, such as online, through activist efforts, and artistic endeavors. Ultimately, there are countless ways that survivors find justice, which points to the need for an expansive and individualized approach.

This review attempted to integrate findings from quantitative, qualitative, and mixed-methods research, as well as reviews, theoretical literature, and commentaries, and further explore how and where survivors enact their justice process. Hence, this review expands the collective understanding of justice beyond a binary understanding linked to an outcome (i.e., justice versus no justice) and complements traditional justice-seeking via the criminal legal system. It also offers other avenues for survivors who feel limited by their preconceived notions of justice, which are informed by what is offered to them by the current systems in place. Table 1 summarizes these findings and implications.

**Table 1.** Critical Findings and Implications.

• Justice after sexual assault is traditionally understood and enacted through the criminal justice system such that the outcomes are binary (i.e., justice is achieved or not achieved) • According to survivors of sexual assault, justice can instead be understood as a dynamic and individualized process that may include the experience of voice, connectedness, participating in a process, accountability, and prevention • The justice process needs to be rooted in safety and control for the survivor • Survivors may seek and enact justice through a myriad of avenues, including criminal justice processes, medical/mental health spaces, activism, art, and social media • Existing systems and actors within these systems, including legal, medical, and mental health personnel should encourage survivors to identify and define their own experience of justice and resist a binary model of justice • Extant systems should, therefore, be flexible and accessible to help survivors realize their preferred modes of justice

A limitation of the current review is that the methods are not reproducible in a manner similar to systematic reviews. We do not contend that this review (i.e., CIS) is all-encompassing of the literature on justice after sexual assault. However, the critical and iterative framework used allowed us to summarize an expansive and idiosyncratic literature base. Because the review requires a significant interpretive and reflexive process, others may read, understand, and summarize the material differently. We hope that our positionality statements clarify and situate the review for the reader. Our work was limited by what is available in the literature base. As most articles were centered on male violence against cis women from a Western perspective of justice, conclusions should not be generalized to *all* women. Looking ahead, it will be important to ask survivors their views on our resultant conceptualization of justice to ascertain its fit and what factors may assist or impede this process. For example, survivors likely experience different barriers to justice, including experiences of marginalization. It would also be a worthy venture to clarify and distinguish the overlapping and conflicting components of justice and healing, as this was a recurring discussion among the research team. Qualitative methods may help to see if and how survivors differentiate these two concepts. Researchers should also consider how to offer opportunities for justice enactment, including feminist-based participatory approaches (Ponic et al., 2010) and critical community-engaged scholarship (Gordon da Cruz, 2018). Finally, efforts are needed to prioritize work with diverse groups of women, including women of color, Indigenous women, sexual minority women, and gender-expansive individuals who are more likely to experience sexual violence (Conroy & Cotter, 2017; Ogrodnik, 2010).

Our model of justice, which we have come to based on the existing literature, has theoretical, clinical, and policy implications. Findings from this review build upon the vital work of activists, scholars, and survivors. Theoretically, our approach aligns with existing models of justice, including kaleidoscopic justice (McGlynn & Westmarland, 2019), Daly’s model of justice (Daly, 2017), and Herman’s writing on justice (Herman, 2023). Namely, there is agreement that conceptualizing justice within a process-based and person-centered approach coheres with perspectives of survivors. While there are different labels for some constructs (e.g., the kaleidoscopic model (McGlynn & Westmarland, 2019) uses “consequences” whereas Herman (2023) and Daly (2017) identify “accountability” as central to justice), there is consensus on their definition.

Differences emerge when comparing the concepts of justice and healing. Some scholars delineate justice and healing in that they are mutually exclusive entities (Daly, 2017; Koss, 2010); this is different from others, such as Herman, who refers to “healing justice” as a holistic term (2023) and Wager who writes about these two concepts in tandem (2013). We did not explore the relation between these concepts as it was beyond the scope of the review; however, this is important in future inquiry. The role of safety, which is central to justice in all components of our model is also identified as a separate entity outside of justice by other scholars (Daly, 2017; Koss, 2010). As such, while there is a large overlap of agreement within the literature, there are also discrepancies and differences between justice models.

In practice, it is important to help survivors, researchers, policymakers, and individuals involved in advocacy to shift their understanding and viewpoint of what justice can be and to move the scope of justice beyond a binary way of thinking. In this way, survivors may experience justice-seeking as empowering, given that there are many options at their disposal rather than be limited by a potentially retraumatizing system (Campbell, 2014). We also found that our justice model necessarily included community support, both for the survivor and the perpetrator, which harkens back to communitarian justice being relevant to sexual violence against women, as described by Koss (2000). Thus, we must contest the larger sociopolitical structures that encourage and facilitate isolation, such as rugged individualism and patriarchy that keep people disconnected. Improved social and community cohesion may facilitate accountability as part of justice in a more meaningful way, as perpetrators and survivors may have higher vested interests in working together toward healing and justice for both of their benefit, which is presently a significant and understandable barrier toward many restorative justice processes (Marsh & Wager, 2015). This was demonstrated by Philly’s PISSED and Philly Stands Up, as Kelly highlighted a shared community as a prerequisite for restorative/transformative justice processes (2011).

Service workers embedded into oft-used systems, such as legal actors, victim advocates, mental healthcare workers, and medical professionals, may benefit from an expanded understanding of justice to reduce hierarchies in justice avenues (i.e., emphasizing criminal justice) and to better align with the wants and needs of survivors. Service workers may also assist survivors in identifying which components of justice are most important for them at the time of seeking support and where they may enact these justice-related behaviors. To do so, workers in traditional systems may have to challenge deeply-held beliefs about justice definitions to be more survivor-centered; this may be implemented by training and workshops that encourage a reflexive practice to challenge beliefs and stereotypes. Funding for community-based programming that provides opportunities for survivors to seek voice, connectedness, participation, accountability, and prevention is warranted. Based on this review, this may include arts-based groups, activism, therapeutic experiences, connecting with nature, and online participatory approaches.

From a psychological process standpoint, it seems important for survivors to experience what has been traditionally referred to as a corrective emotional experience (CEE) as part of their justice experience. CEE was originally introduced by Alexander and French (1946, p. 338) as a therapeutic process that involves “reexperiencing the old, unsettled conflict but with a new ending.” Indeed, this is what survivors are seeking in the response from others, including their community and potential systems, following a sexual assault; that is, a different outcome than the trauma. For example, instead of being overpowered, silenced, and degraded, a CEE might include being treated with respect, being empowered, and feeling heard by others. This is important because of the common experience of retraumatization when seeking justice through traditional legal pathways, in which the trauma is often re-enacted. Clinicians can use our framework to help clients identify and enact justice actions salient to them; clinicians may incorporate psychoeducation about CEE as well as provide these experiences within the therapeutic relationship. In research, it will be important to continue using idiographic and community-based approaches when learning *with* survivors.

Prevention work needs to be intersectionally-informed. There is considerable and excellent work being done at university campuses. However, this sometimes means that research focuses on young, white cis women (Cadaret et al., 2021; Hahn et al., 2017; Jozkowski, 2015). It will be important to continue to fund and disseminate this prevention work to other community-based groups as well as earlier in the education system. In doing so, prevention work should be culturally responsive, involve community leaders and families, and meet youth where they are. This may occur in educational settings but may also include faith-based locations, sports programming, and camps (Baldwin-White & Moses, 2021; Hubach et al., 2019; Khoori et al., 2020; Yu et al., 2017). Survivors can be involved as part of their justice journey.

In conclusion, justice for survivors of sexual assault may be understood as a dynamic and iterative process that must be grounded in safety and control. The experience of justice is not finite, which, for some survivors, is counter to the criminal legal model. Expanding the cultural definition of justice may empower survivors of sexual assault to clarify and seek their own experiences of justice rather than feel limited to a binary approach propagated by the present systems in place.

## Supplemental Material

sj-docx-1-tva-10.1177_15248380241248411 – Supplemental material for Justice for Women After Sexual Assault: A Critical Interpretive Synthesis Supplemental material, sj-docx-1-tva-10.1177_15248380241248411 for Justice for Women After Sexual Assault: A Critical Interpretive Synthesis by Joanna Collaton, Paula Barata, Mavis Morton, Kim Barton and Stephen P. Lewis in Trauma, Violence, & Abuse
