# Supplementary material for: Justice for Women After Sexual Assault: A Critical Interpretive Synthesis
Source: Trauma Violence Abuse. 2024 May 9;25(5):3457–72. doi: 10.1177/15248380241248411 (PMC11545202; doi:10.1177/15248380241248411)
Supplement: sj-docx-1-tva-10.1177_15248380241248411 – Supplemental material for Justice for Women After Sexual Assault: A Critical Interpretive Synthesis [file sj-docx-1-tva-10.1177_15248380241248411.docx]

Appendix A

List of websites consulted to identify grey literature:

1. LEAF – Women’s Legal Education and Action Fund (https://www.leaf.ca/)
2. Women’s College Hospital SA/DVCC (https://www.womensresearch.ca/gender-based-violence/)
3. AWID (https://www.awid.org/)
4. NNEDV (https://nnedv.org/)
5. Women’s Aid (https://www.womensaid.ie/)
6. Imkaan (https://www.imkaan.org.uk/resources)
7. Project Restore (https://www.projectrestore.nz/)
8. Women and Girls network (https://www.wgn.org.uk/)
9. ANROWS (https://www.anrows.org.au/publications/rtpp/)
10. National Center on Domestic and Sexual Violence (https://www.nsvrc.org/)
11. Rape Crisis Network Ireland (https://www.rcni.ie/)
12. Rape Crisis England and Wales (https://rapecrisis.org.uk/)
13. UN Inventory of VAW initiatives (https://evaw-un-inventory.unwomen.org/en/agencies/un-women)
14. IWPR (https://iwpr.org/)
